# Susceptibility of Ocular *Staphylococcus aureus* to Antibiotics and Multipurpose Disinfecting Solutions

**DOI:** 10.3390/antibiotics10101203

**Published:** 2021-10-03

**Authors:** Madeeha Afzal, Ajay Kumar Vijay, Fiona Stapleton, Mark D. P. Willcox

**Affiliations:** School of Optometry and Vision Science, University of New South Wales, Sydney 2052, Australia; v.ajaykumar@unsw.edu.au (A.K.V.); f.stapleton@unsw.edu.au (F.S.)

**Keywords:** *Staphylococcus aureus*, microbial keratitis, conjunctivitis, corneal infiltrative events, antibiotic susceptibility, MPDS susceptibility

## Abstract

*Staphylococcus aureus* is a frequent cause of ocular surface infections worldwide. Of these surface infections, those involving the cornea (microbial keratitis) are most sight-threatening. *S. aureus* can also cause conjunctivitis and contact lens-related non-infectious corneal infiltrative events (niCIE). The aim of this study was to determine the rates of resistance of *S. aureus* isolates to antibiotics and disinfecting solutions from these different ocular surface conditions. In total, 63 *S. aureus* strains from the USA and Australia were evaluated; 14 were from niCIE, 26 from conjunctivitis, and 23 from microbial keratitis (MK). The minimum inhibitory (MIC) and minimum bactericidal concentrations (MBC) of all the strains to ciprofloxacin, ceftazidime, oxacillin, gentamicin, vancomycin, chloramphenicol, azithromycin, and polymyxin B were determined. The MIC and MBC of the niCIE strains to contact lens multipurpose disinfectant solutions (MPDSs) was determined. All isolates were susceptible to vancomycin (100%). The susceptibility to other antibiotics decreased in the following order: gentamicin (98%), chloramphenicol (76%), oxacillin (74%), ciprofloxacin (46%), ceftazidime (11%), azithromycin (8%), and polymyxin B (8%). In total, 87% of all the isolates were multidrug resistant and 17% of the isolates from microbial keratitis were extensively drug resistant. The microbial keratitis strains from Australia were usually susceptible to ciprofloxacin (57% vs. 11%; *p* = 0.04) and oxacillin (93% vs. 11%; *p* = 0.02) compared to microbial keratitis isolates from the USA. Microbial keratitis isolates from the USA were less susceptible (55%) to chloramphenicol compared to conjunctivitis strains (95%; *p* = 0.01). Similarly, 75% of conjunctivitis strains from Australia were susceptible to chloramphenicol compared to 14% of microbial keratitis strains (*p* = 0.04). Most (93%) strains isolated from contact lens wearers were killed in 100% MPDS, except *S. aureus* 27. OPTI-FREE PureMoist was the most active MPDS against all strains with 35% of strains having an MIC ≤ 11.36%. There was a significant difference in susceptibility between OPTI-FREE PureMoist and Biotrue (*p* = 0.02). *S. aureus* non-infectious CIE strains were more susceptible to antibiotics than conjunctivitis strains and conjunctivitis strains were more susceptible than microbial keratitis strains. Microbial keratitis strains from Australia (isolated between 2006 and 2018) were more susceptible to antibiotics in comparison with microbial keratitis strains from the USA (isolated in 2004). Most of the strains were multidrug-resistant. There was variability in the susceptibility of contact lens isolates to MPDSs with one *S. aureus* strain, *S. aureus* 27, isolated from niCIE, in Australia in 1997 being highly resistant to all four MPDSs and three different types of antibiotics. Knowledge of the rates of resistance to antibiotics in different conditions and regions could help guide treatment of these diseases.

## 1. Introduction

*S. aureus* is one of the most common causes of ocular infections worldwide [1]. It has been reported as the most common cause of microbial keratitis (MK), which is a sight-threatening infection of the cornea [2] in Australia [3,4] and the USA, [5,6]. Conjunctival infection (conjunctivitis) is also frequently caused by *S. aureus* [7]. *S. aureus* is also commonly observed in inflammatory adverse reactions associated with contact lens-wearing. These corneal infiltrative events are differentiated into infections or inflammatory conditions; the latter are collectively called non-infectious corneal infiltrative events (niCIE) [8].

Treatment of MK involves the intensive use of topical antibiotics and commonly monotherapy with fluoroquinolones or with the use of fortified antibiotics (for example, a beta lactam such as cefazolin with an aminoglycoside such as tobramycin or gentamicin) [9,10]. Conjunctivitis may be treated by topical application of tetracycline, chloramphenicol, or fluoroquinolones [11]. Conversely non-infectious corneal infiltrative events (niCIEs) are self-limiting and heal upon removal of the contact lens, although prophylactic treatment with topical broad-spectrum antibiotics such as fluoroquinolones, chloramphenicol, and polymyxin B with low dose topical steroids [8] may be used.

*S. aureus* infections can be difficult to treat because strains may be resistant to multiple antibiotics. *S. aureus* can acquire resistance to virtually every antibiotic that has entered clinical use [12]. Bacteria have developed sophisticated mechanisms of drug resistance to ensure their survival. Resistance to antibiotics can be achieved through multiple biochemical pathways [13] that include modification [14] and destruction of antibiotic molecules [15], decreased antibiotic penetration or increased efflux [16,17,18], modification or complete replacement, or bypassing of target site [19,20]. The effects of various antibiotics on cytoplasmic peptidoglycan metabolite levels in MRSA were determined and metabolite levels were high in *S. aureus* [21]. Increasing antimicrobial resistance of *S. aureus* has been identified as a public health threat by the World Health Organization [22]. Since emerging in 1961, the incidence and prevalence of methicillin-resistant *S. aureus* (MRSA) in ocular infections has increased dramatically [23,24]. Antibiotic resistance in *S. aureus* can be both inherited and acquired. Inherited resistance [25] includes genes naturally present on chromosomes which confer low membrane permeability, efflux pump expression, and enzymatic inactivation of antibiotics [26]. Acquired resistance includes genetic mutations [27] and horizontal transfer of genes across the strains via mobile genetic elements [28].

Contact lens multipurpose disinfectant solutions (MPDS) are used to disinfect contact lenses when they are not being worn. MPDSs contain disinfectants such as quaternary ammonium compounds or biguanides. *S. aureus* strains which possess *qac* genes can be resistant to disinfectants and are more commonly resistant to antibiotics [22]. As *qac* genes occur alongside genes for antibiotic resistance, there is concern that resistance to disinfectants may increase the spread of antibiotic resistance [29].

There is limited information available on antimicrobial and MPDS susceptibility patterns of clinical isolates of *S. aureus* from Australia in comparison to other countries. The purpose of this study was to investigate the antibiotic and MPDS sensitivities of *S. aureus* isolates from different ocular surface conditions isolated in Australia and the USA.

## 2. Results

### 2.1. Antibiotic Susceptibilities

Table 1 summarizes the MIC and MBC of *S. aureus* strains to antibiotics. All isolates were susceptible to vancomycin (100%). The susceptibility to the other antibiotics decreased in the following order: gentamicin (98%), chloramphenicol (76%), oxacillin (74%), ciprofloxacin (46%), ceftazidime (11%), azithromycin (8%), and polymyxin B (8%). Most of the microbial keratitis strains from Australia (isolated between 2006 and 2018) were more commonly susceptible to ciprofloxacin (57%) and oxacillin (93%) compared to microbial keratitis strains from the USA (isolated in 2004) for ciprofloxacin (11%; *p* = 0.04) and oxacillin (11%; *p* = 0.02).

Chloramphenicol susceptibility varied by ocular condition and origin of the isolates. In total, 95% of conjunctivitis (isolated in 2006) and 78% of non-infectious CIE strains (isolated between 1995 and 2001) from Australia were susceptible to chloramphenicol. There was a significantly lower rate of susceptibility of microbial keratitis strains from Australia (14%) compared to Australian conjunctivitis strains (95%; *p* = 0.04). There was a similar pattern amongst the USA isolates (isolated in 2004), with 55% of the microbial keratitis strains and 95% of the conjunctivitis strains being sensitive to chloramphenicol. Overall, 30% of microbial keratitis strains from Australia (isolated between 2006 and 2018) and the USA (isolated in 2004) were susceptible to chloramphenicol rather than conjunctivitis (isolated between 2004 and 2006) or non-infectious CIE strains (85%; *p* = 0.01).

Most strains (87%; 55/63) were multidrug-resistant (MDR), which is defined as being resistant to three different classes of antibiotics [22]. Strains 111, 112, and 113 from the USA (microbial keratitis; isolated in 2004) and M43-01 from the Australian (microbial keratitis; isolated in 2018) group (see Table S1, Supplementary Material) were extensively drug-resistant (XDR) strains, which is defined as resistant to almost all antibiotics classes [30]. Strain 32 from Australia (niCIE; isolated in 1997) and strain 46 from the USA (conjunctivitis; isolated in 2004) were susceptible to all antibiotics used. Strains from niCIE (isolated between 1995 and 1999) were more susceptible to antibiotics compared to strains from infections (conjunctivitis + microbial keratitis; isolated between 2004 and 2018). The susceptibility of microbial keratitis strains varied by origin of isolates, with microbial keratitis *S. aureus* strains from the USA being more likely to be MRSA and multidrug-resistant compared to Australian microbial keratitis strains.

### 2.2. Multipurpose Solution Susceptibility

Isolates from contact lens-related niCIE (isolated between 1995 and 2001) were tested for their susceptibility to the MPDSs. All MPDSs showed good activity against the isolates when used at 100% concentration. After diluting the MPDS, strains were able to grow at different dilutions. Overall, OPTI-FREE PureMoist had the lowest median, namely a median MIC of 5.64% and a median MBC of 11.36%, followed by the Renu Advanced Formula (median MIC of 11.36% and median MBC of 22.72%). Complete RevitaLens OcuTec and Biotrue had similar median MICs of 22.72% and median MBCs of 45.45% (Table 2). There was a significant difference in the MIC between OPTI-FREE PureMoist and Biotrue (*p* = 0.02), where strains were more likely to be resistant to Biotrue. One MDR strain (*S. aureus* 27; isolated in 1997) had a relatively high MIC and MBC, of >90%, compared to Biotrue and Renu Advanced Formula, and moderately high levels for OPTI-FREE PureMoist and Complete RevitaLens Ocutec. The MBCs for all the MPDSs were usually twice the MICs.

### 2.3. Antibiotic and MPDS Susceptibility of niCIE Strains

Bacterial strains can be described as susceptible or resistant to an antibiotic; however, there is no such definition for MPDS in the literature. A previous study [31] categorized strains with a MIC greater than 10% as resistant to MPDSs and this classification was used in the current study. While the 10% cut-off used seems arbitrary, it is useful to demonstrate the consequences of improper use of MPDSs during contact lens-wearing as the practice of topping off and reusing MPDSs is a risk factor for infection for contact lens-wearers [32,33]; thus, it is useful to model the consequences of improper use of MPDSs. There was no concordance between antibiotic and MPDS sensitivity (Table 3), thus antibiotic sensitivity was not a good predictor of resistance to MPDSs. One strain (*S. aureus* 27) was resistant to four out of the eight antibiotics and to all MPDSs. Conversely, the strains *S. aureus* 28 and 33, isolated in 1997, were susceptible to six out of the eight antibiotics, while being resistant to all MPDSs (Table 3).

## 3. Discussion

This study reports the in vitro susceptibility of ocular strains of *Staphylococcus aureus* from the USA and Australia to commonly used antibiotics and the susceptibility of some strains to contact lens MPDSs. Microbial keratitis strains from Australia (isolated between 2006 and 2018) were more commonly sensitive to fluoroquinolones and oxacillin than the strains from the USA (isolated in 2004). Differences in the antibiotic susceptibility profiles in different geographical populations is not uncommon and may be due to climate [34] or cultural differences [35,36,37,38]. One study has shown that widespread over-the-counter supply of antibiotics can underpin high resistance [39] and the ability to access antibiotics in such a way differs between countries.

All strains were susceptible to vancomycin, at 100%, and gentamicin, at 98%. Vancomycin resistance in systemic infections has been reported, [40] however, no resistance has been reported in ocular isolates [4]. Gentamicin is commonly prescribed in *S. aureus* ocular infections, but its susceptibility rates vary [41]. The current results are consistent with other studies from the USA and Australia for *S. aureus* ocular isolates [10,42,43,44]. The antibiotic susceptibility profile in the current study suggests gentamicin to be the best option to treat *S. aureus* ocular infections in both Australia and the USA, and vancomycin to be reserved to treat isolates that are resistant to other antibiotics.

Overall, less than half (46%; 29/63) of all the strains in the current study were sensitive to ciprofloxacin. Studies from Australia published between 2014 and 2016 reported that 93 to 100% of microbial keratitis isolates were susceptible to ciprofloxacin [45,46,47,48]. In contrast, the current study reports increasing resistance of *S. aureus* strains from Australia to ciprofloxacin (66%). The increasing rate of ciprofloxacin resistance in Australian microbial keratitis strains (isolated between 2006 and 2018) is of concern, as fluoroquinolones are the first line of treatment for keratitis in Australia [4]. It would be important to explore this in a larger study. Similarly, the rate of resistance of the USA ocular *S. aureus* isolates (isolated in 2004) to ciprofloxacin in the current study was higher than in Australia. One possible reason is that in Australia, antibiotic use in animals is restricted compared to other countries, including the USA [49], which may account for the low level of resistance of Australian isolates. It is generally believed that bacteria that infect the eye are derived from a general pool of environmental bacteria. Resistant bacteria are transmitted to humans through direct contact with animals [50], through the environment [51], and through food products [52]. The increasing antibiotic resistance worldwide has been attributed to their widespread systemic use, their over-the-counter availability, and their inappropriate use [53] in agriculture and veterinary practices to promote growth and prevent infections in livestock [54,55]. Similarly, in ocular infections, factors such as empirical prescription, short-term exposure, and repeated exposure of antibiotics contributed to the resistance of ocular pathogens [56] and changes in resident ocular flora [57]. A large surveillance study from the USA on the antibiotic resistance among ocular isolates between 2009 and 2016 found that approximately 36% of the ocular *S. aureus* isolates were resistant to ciprofloxacin [58]. An increased proportion of MRSA, from 8.5% to 27.9%, in *S. aureus* isolates collected between 1990 and 2001, has been reported in the USA [59]. MRSA strains are often resistant to fluoroquinolones [58,59,60,61]. However, in the current study, only 7% of MRSA strains from Australia (isolated between 2006 and 2018) were ciprofloxacin-resistant, whereas 78% of MRSA strains from the USA (isolated in 2004) were resistant to ciprofloxacin, which is consistent with a previous report from the USA [58]. The mechanism of resistance of ocular MRSA strains resistant to ciprofloxacin is unclear and requires further study.

In the current study, only 11% of *S. aureus* strains were susceptible to ceftazidime and all microbial keratitis strains were resistant to this antibiotic. An increasing rate of resistance of *S. aureus* microbial keratitis isolates to first-generation cephalosporins (cephalothin) over a period of 15 years has been reported [62]. Ceftazidime is generally reported to be active against *S. aureus*, except MRSA strains, but it is less active against *S. aureus* than first and second-generation cephalosporins [63]. Resistance to ceftazidime, a third-generation cephalosporin which can be used to treat MRSA, is horizontally acquired due to β-lactamases or due to alteration and over-expression of the penicillin binding protein [64]. In the current study, the mechanism of resistance may have been different depending on the disease or the country from which the strains were isolated.

In the present study, chloramphenicol remained as a good choice of treatment for conjunctivitis and niCIE caused by *S. aureus*, as 96% and 78% of isolates, respectively, were susceptible. Gram-positive bacteria isolated from microbial keratitis isolates have also been reported regarding low levels of chloramphenicol resistance in the Australian and USA isolates [65,66]. However, the current study findings of the increasing resistance of microbial keratitis strains from Australia [67], isolated between 2006 and 2018, and from the USA (45%), isolated in 2004, are not consistent with these earlier studies and suggest it is a poor choice for treatment of corneal infections. Resistance to chloramphenicol may be inherited [68,69,70] or acquired [71,72,73]. The underlying mechanism for the difference in chloramphenicol susceptibility between infectious (MK+ conjunctivitis) and non-infectious ocular conditions requires further investigation.

Most of the *S. aureus* strains in the current study were resistant to azithromycin. Most of the resistant strains were also MRSA, which supports the results of a previous study [10], and most of the strains were resistant to polymyxin B. Polymyxin B is considered a Gram-negative antibiotic that does not diffuse well in mediums and resistance to this antibiotic is characteristic of *S. aureus* [74]. This study supports previous recommendations that Polymyxin B is not a good choice for the treatment of *S. aureus* ocular infections [75].

Only 6% of Australian strains (2/32), isolated between 2006 and 2018, were resistant to oxacillin (i.e., could be classified as MRSA), and conversely, 45% of all the USA strains (14/31), isolated in 2004, were resistant to oxacillin. In the USA, an increase in the proportion of MRSAs among *S. aureus* ocular isolates, specifically from 29.5% in 2000 to 41.6% in 2005, has been reported in a national surveillance study (ARMOR) [10]. The high level of MRSAs among *S. aureus* isolates is of concern as MRSA is believed to cause more severe diseases than methicillin-sensitive *S. aureus* [76]. Further molecular analysis of the geographical variation of MRSA in the USA and of the Australian microbial keratitis and conjunctivitis strains, as well as of community or hospital-acquired MRSA, is required.

The study has demonstrated that niCIE strains of *S. aureus*, isolated between 1995 and 2001, vary in their susceptibilities to MPDSs. Most of the strains were susceptible to all MPDSs when used at 100% concentrations, indicating a good activity of the MPDSs. The most effective MPDS, specifically OPTI-FREE PureMoist, contains two disinfectants, namely Polyquaternium-1 and Aldox. Polyquaternium-1 showed good activity against *S. aureus* when used alone, as Aldox has been shown to do, as well [77]. Renu Advanced was the second most effective MPDS in the current study. It contains three disinfectants, namely alexidine, PAPB, and polyquaternium-1. All these disinfectants have been reported to be effective against bacteria [77,78,79,80] and some against their biofilms [81].

Complete RevitaLens, containing alexidine and Polyquaternium, was the third most effective MPDS against *S. aureus* isolates in the present study, but has also been reported to show equal efficacy to OPTI-FREE against *S. aureus* in a previous study [82]. Even though both the disinfectants are effective against *S. aureus* [77,80], dilution of the MPDS decreased its efficacy. Biotrue was the least effective MPDS against *S. aureus* isolates in the current study. Biotrue contains only polyaminopropyl biguanide (PAPB, which is also known as polyhexamethylene biguanide (PHMB). PAPB is active against *S. aureus* [83] but its efficacy is concentration-dependent [84]. One study reported a reduced concentration of PAPB (PHMB) after soaking contact lenses in Biotrue and this lower concentration was associated with its decreased antimicrobial activity against *S. aureus* [84]. The findings of the current study regarding the most to least active MPDSs against *S. aureus* are, in general, in agreement with another study [84].

Resistance to disinfectants can be mediated by the *qac* gene, which can be carried on the same transmissible elements as antibiotic resistance genes [67,84]. While possession of *qac* has been associated with resistance to antibiotics [84], there was no clear phenotypic relationship between antibiotic and MPDS resistance observed in the current study. These strains have not been genotyped previously and exploring whether these strains possess the *qac* gene would help to understand the genotypic relationship between antibiotic and MPDS resistance. Other issues could be addressed in future studies by exploring the biocides in the MPDS as well as their dilutions and effects on MIC individually and in combination with other biocides.

The current study used a convenience sample of strains within the culture collection. All strains from the USA were isolated in 2004. Surveillance studies have shown that the rates of the methicillin resistance of *S. aureus* isolated from keratitis in the USA has not changed from 1997 to 2012 [41]. Overall antibiotic susceptibility has shown little or no change in the resistance patterns of ocular *S. aureus* over the periods of 2009–2013 [85] and 2009–2016 [10]. Similarly, strains isolated from keratitis in Australia between 2005 and 2015 showed little or no change in antibiotic susceptibility to ceftazidime, gentamicin, chloramphenicol, fluoroquinolones, and vancomycin [4]. This panel of antibiotics were used in the current study. The Australian strains used in this study were isolated between 1995 and 2018, with the majority from infection isolated between 2006 and 2018 (17/18). Understanding the susceptibility pattern of these strains could help to reduce the risk of inappropriate antimicrobial prescribing. However, as resistance rates can change over time, future studies should examine strains isolated within matched timeframes. Another issue that could be addressed in future studies is whether the use of combinations of antibiotics can overcome any of the resistance observed.

## 4. Materials and Methods

### 4.1. Staphylococcus aureus Isolates

In total, 63 *S. aureus* clinical isolates were evaluated (Table 4). Strains from the Bascom Palmer Institute, Miami (USA), were kindly provided by Dr Darlene Miller, while those from the Prince of Wales Hospital (Australia) were kindly provided by Dr. Monica Lahra. All strains were stored in culture collection at the School of Optometry and Vision Science, UNSW. The identity of the strains was confirmed using the automated identification system VITEK 2 for Gram-positive bacteria (BioMérieux, Baulkham Hills, NSW, Australia) according to the manufacturer’s instructions.

### 4.2. Susceptibility to Antibiotics

The susceptibility of *S. aureus* strains to different antibiotics was assessed according to the standard protocol described by the Clinical and Laboratory Institute [86]. Antibiotics commonly used to treat these ocular conditions in Australia and in the USA were selected for the test panel and antibiotic stock solutions were prepared following the manufacturer’s recommendations. Antibiotics were diluted in Mueller-Hinton II broth (cation-adjusted, Becton Dickinson and Company, Franklin Lakes, NJ, USA) in sterile 96-well plates to provide the final concentrations ranging from 5120 µg/mL to 0.25 µg/mL.

Bacterial cells at a final concentration of 1 × 10^5^ CFU/mL were then inoculated into 96-well plates with different dilutions of antibiotics and incubated at 37 °C for 18–24 h. For Oxacillin and Vancomycin MIC, *S. aureus* strains were incubated at 35 °C according to CLSI standards [86]. Growth turbidity was measured using a spectrophotometer (FLUOstar Omega, BMG LABTECH, Ortenberg, Germany) at 660 nm. The MIC was taken as the lowest concentration of an antibiotic with no visible growth. For minimum bactericidal concentration (MBC), viable counts were performed by subculturing the cells onto Mueller-Hinton agar (Becton Dickinson and Company, Franklin Lakes, NJ, USA) at their MIC and at the next two higher dilutions of antibiotics; afterwards, they were incubated at 37 °C for 18–24 h. The MBC was the concentration of antibiotics that showed 99.99% bacterial killing [87,88]. The results were interpreted using breakpoints from the Clinical and Laboratory Standards Institute [86] and the European Committee on Antimicrobial Susceptibility Testing [88]. Both resistant and intermediate resistant strains were considered resistant for the subsequent analyses.

### 4.3. Susceptibility to Multipurpose Disinfectant Solutions

Susceptibility of the bacterial strains isolated from contact lens-related niCIE to four commercially available MPDSs (Table 5) was assessed. This testing was restricted to these isolates as all other strains were isolated from non-contact lens-wearers. The MPDSs were OPTI-FREE PureMoist (Alcon, Fort Worth, TX, USA), Complete RevitaLens OcuTec (Abbot Medical Optics, Hangzhou, China), and Biotrue and Renu Advanced Formula (Bausch + Lomb, Rochester, NY, USA; Table 5). MPDS susceptibility was tested using previously published methods [31,85]. In brief, each MPDS was serially diluted in freshly prepared sterile phosphate-buffered saline (NaCl 80 g/L, Na_2_HPO_4_ 11.5 g/L, KCl 2 g/L, and KH_2_PO_4_ 2 g/L, pH = 7.2) to protect the bacteria from pH shock. The serially diluted MPDS (200 µL) was added to wells of a microtiter plate and a 20 µL bacterial suspension was added to achieve a final concentration of 1 × 10^5^ CFU/mL. Positive (PBS + bacteria) and negative controls (undiluted PBS) were used. The plates were incubated at 37 °C for 18–24 h. Growth turbidity was measured using a spectrophotometer (FLUOstar Omega, BMG LABTECH, Germany) at 660 nm. Strains with a MIC of more than 10% MPDS were considered resistant. MBC was the concentration of the MPDS that gave 99.99% (3 log units) bacterial killing [85,89]. The purpose of testing MPDSs outside the stated instruction was to find the MIC of *S. aureus* that caused corneal infiltrative events, as concentrations of disinfectants through topping off or through the reuse of disinfecting solutions have been identified as a risk factor for contact lens-related corneal infections [33]. There is some evidence that this may occur more frequently with certain MPDS products, thus it is not unreasonable to challenge MPDS products in a way that may mimic their use in the community.

### 4.4. Statistical Analysis

Differences in the frequency of antibiotic susceptibility between infectious (MK+ conjunctivitis) and non-infectious (niCIE) groups from Australia and the USA, and MPDS susceptibility in contact lens-related niCIE strains were only compared using Fisher’s exact test (GraphPad prism, 2019, v8.0.2.263). For all analyses, a *p*-value of <0.05 was considered statistically significant.

## 5. Conclusions

This study concludes that *S. aureus* strains isolated from microbial keratitis from the USA (isolated in 2004) were more likely to be MRSA and multidrug-resistant compared to Australian microbial keratitis strains (isolated between 2006 and 2018). In addition, microbial keratitis strains from the USA and Australia were less susceptible to antibiotics compared to conjunctivitis (isolated in 2004–2006) and non-infectious CIE strains (isolated between 1995 and 2001). Exploring the genomic resistance mechanisms and possession of virulence traits between infectious (MK+ conjunctivitis) and non-infectious ocular conditions from the USA and Australia may help to understand these susceptibility findings. The findings of this study will help to understand the resistance pattern of ocular *S. aureus* isolates from the USA and Australia, which will further inform treatment options.

## Figures and Tables

**Table 1:** Percentage of sensitivity and resistance of *S. aureus* strains from different ocular conditions to antibiotics.

Antibiotic	Microbial Keratitis (*n* = 23)		Conjunctivitis (*n* = 26)		niCIE (*n* = 14)	
	% S	% R	% S	% R	% S	% R
Ciprofloxacin	39.1	60.8	42.3	57.6	71.4	28.5
Ceftazidime	0	100	11.5	88.4	28.5	71.4
Oxacillin	60.8	39.1	76.9	23	92.8	7.1
Gentamicin	95.6	4.3	100	0	100	0
Vancomycin	100	0	100	0	100	0
Chloramphenicol	30.4	69.5	92.3	7.6	78.5	21.4
Azithromycin	0	100	15.3	84.6	7.1	92.8
Polymyxin B	0	100	15.3	84.6	7.1	92.8

Abbreviations: R = resistant; S = susceptible; and niCIE = non-infectious corneal infiltrative events.

**Table 2:** Minimum inhibitory concentration of MPDSs for *S. aureus* niCIE isolates associated with contact lenses.

*S. aureus* Strains	OPTI-FREE PureMoist (%)		Renu Advanced Formula (%)		Complete RevitaLens OcuTec (%)		Biotrue (%)	
	MIC	MBC	MIC	MBC	MIC	MBC	MIC	MBC
12	2.84	11.36	2.84	5.64	2.84	5.64	11.36	22.72
20	11.36	22.72	11.36	22.72	22.72	22.72	45.45	90.9
24	5.64	11.36	2.84	11.36	45.45	90.9	11.36	22.72
25	1.42	2.84	1.42	5.64	2.84	5.64	5.64	11.36
26	1.42	5.64	1.42	2.84	5.64	11.36	22.72	45.45
27	22.72	22.72	90.9	90.9	22.72	45.45	90.9	90.9
28	11.36	22.72	11.36	22.72	22.72	45.45	45.45	90.9
29	5.64	11.36	22.72	45.45	22.72	45.45	45.45	90.9
31	11.36	22.72	22.72	45.45	22.72	45.45	5.64	11.36
32	5.64	11.36	22.72	45.45	22.72	45.45	22.72	45.45
33	11.36	22.72	22.72	45.45	22.72	45.45	45.45	90.9
41	5.64	11.36	11.36	45.45	11.36	45.45	22.72	45.45
48	2.84	5.64	2.84	5.64	2.84	5.64	5.64	11.36
117	11.36	22.72	5.64	22.72	11.36	11.36	11.36	22.72

**Table 3:** Relative susceptibilities of contact lens-related niCIE isolates to antibiotics and MPDSs.

Strains	ANTIBIOTICS								MPDS			
	CIP	CEFT	OXA	GEN	VAN	CHL	AZI	P-B	OPTI	RENU	REV	BIO
12												
20												
24												
25												
27												
28												
32												
33												
48												
117												
26												
29												
31												
41												

No shading indicates that strains were susceptible, and gray indicates they were resistant. Abbreviations: CIP, Ciprofloxacin; CEFT, Ceftazidime; OXA, Oxacillin; GEN, Gentamicin; VAN, Vancomycin; CHL, Chloramphenicol; AZI, Azithromycin; P-B, Polymyxin B; OPTI, OPTI-FREE PureMoist; RENU, Renu Advanced Formula; REV, Complete RevitaLens OcuTec; and BIO, Biotrue.

**Table 4:** *S. aureus* ocular isolates used in the study.

*S. aureus* Isolates	Origin	Associated Condition	Year of Isolation
106	Bascom Palmer Institute, Miami (USA)	Microbial keratitis (MK)	2004
107			
108			
109			
110			
111			
112			
113			
114			
129	Prince of Wales Hospital (Australia)		2006
34			1997
M5-01			2018
M19-01			
M27-01			
M28-01			
M30-01			
M36-01			
M43-01			
M49-02			
M65-02			
M71-01			
M90-01			
M91-01			
84	Bascom Palmer Institute, Miami (USA)	Conjunctivitis	2004
85			
86			
87			
88			
89			
90			
91			
92			
93			
94			
95			
96			
97			
98			
99			
100			
101			
102			
103			
104			
105			
46	Prince of Wales Hospital (Australia)		2006
134			
136			
140			
12	SOVS, UNSW (Australia)	Contact lens-related non-infectious corneal infiltrative events (niCIE)	1995
20			
24			1996
25			
26			
27			1997
28			
29			
31			
32			
33			
41			1999
48			2001
117			1999

**Table 5:** Multipurpose disinfecting solutions and their active agents.

MPDS	Manufacturer	Disinfectants and Their Concentrations
OPTI-FREE^®^ PureMoist^®^	Alcon, Fort Worth, TX, USA	Polyquaternium-1, 10 ppm; Aldox, 6 ppm
Complete RevitaLens OcuTec (now sold as ACUVUE™ RevitaLens)	Abbot Medical Optics, Hangzhou, ZJ, China (Johnson and Johnson Vision)	Alexidine dihydrochloride, 1.6 ppm; polyquaternium-1, 3 ppm
Biotrue^®^	Bausch + Lomb, Rochester, NY, USA	Polyaminopropyl biguanide, 1.3 ppm; polyquaternium-1, 1 ppm
Renu^®^ Advanced Formula		Polyaminopropyl biguanide, 0.5 ppm; polyquaternium-1, 1.5 ppm; alexidine, 2 ppm

## Data Availability

Data supporting this study is available in the supplementary material.

## Supplementary Materials

The following are available online at https://www.mdpi.com/article/10.3390/antibiotics10101203/s1, Table S1: Details of the MIC and MBC of *S. aureus* strains from different ocular conditions to the antibiotics used in the current study.
