# Supplementary material for: Susceptibility of Ocular Staphylococcus aureus to Antibiotics and Multipurpose Disinfecting Solutions
Source: Antibiotics (Basel). 2021 Oct 3;10(10):1203. doi: 10.3390/antibiotics10101203 (PMC8533015; doi:10.3390/antibiotics10101203)
Supplement: Supplementary file 1 [file antibiotics-10-01203-s001.zip › antibiotics-1378324-supplementary.pdf]

Table S1. MIC and MBC of *S. aureus* strains from different ocular conditions to antibiotics.

Ocular condition	Strains	Ciprofloxacin ≤1, 2, ≥ 4* μg/ml		Ceftazidime ≤8, 16, ≥ 32 μg/ml		Oxacillin ≤ 2, ≥ 4 μg/ml		Gentamicin ≤4, 8, ≥16 μg/ml		Vancomycin ≤2, 4-8, ≥16 μg/ml		Chloramphenicol ≤8, 16, ≥ 32 μg/ml		Azithromycin ≤2, 4 ≥ 8 μg/ml		Polymyxin B ≤2, 4, ≥ 8	
		MIC	MBC	MIC	MBC	MIC	MBC	MBC	MBC	MIC	MBC	MIC	MBC	MIC	MBC	MIC	MBC
Microbial keratitis USA	106	128 (R)	256	128 (R)	256	8 (R)	16	1 (S)	2	0.5 (S)	1	8 (S)	16	16 (R)	32	40 (R)	16
	107	64 (R)	128	64 (R)	128	128 (R)	256	0.5 (S)	1	0.5 (S)	1	16 (S)	32	16 (R)	32	160 (R)	32
	108	1 (S)	2	64 (R)	128	32 (R)	32	1 (S)	2	0.5 (S)	1	8 (S)	16	8 (R)	16	4 (R)	8
	109	128(R)	256	128 (R)	256	128 (R)	256	1 (S)	2	0.5 (S)	1	8 (S)	16	16 (R)	32	4 (I)	8
	110	128(R)	256	64 (R)	128	128 (R)	256	0.5 (S)	1	0.5 (S)	1	8 (S)	16	128(R)	256	80 (R)	16
	111	1280 (R)	2560	32 (R)	64	128 (R)	256	8 (I)	16	2 (S)	2	32 (R)	64	128 (R)	256	80 (R)	16
	112	2560 (R)	5120	32 (R)	64	128 (R)	256	2 (S)	4	1 (S)	2	32 (R)	64	128 (R)	256	320 (R)	640
	113	1280 (R)	2560	32 (R)	64	32 (R)	64	1 (S)	2	1 (S)	1	32 (R)	64	320(R)	1280	4 (I)	8
	114	8 (R)	16	16 (I)	32	0.5 (S)	1	4 (S)	8	0.5 (S)	1	32 (R)	64	640 (R)	1280	8 (R)	16
Microbial keratitis AUS	34	1 (S)	2	64 (R)	64	1 (S)	1	1 (S)	2	1 (S)	1	8 (S)	16	16 (R)	32	128 (R)	256
	129	1 (S)	1	16 (I)	32	0.5 (S)	1	0.5 (S)	1	2 (S)	2	32 (R)	64	32 (R)	64	40 (R)	16
	M5-01	64 (R)	128	128 (R)	256	2 (S)	4	2 (S)	2	1 (S)	2	128 (R)	256	8 (R)	16	128 (R)	256
	M19-01	1 (S)	2	128(R)	256	0.5 (S)	1	2 (S)	2	1 (S)	2	2 (S)	4	16 (R)	32	128 (R)	256
	M27-01	1 (S)	2	128 (R)	256	0.5 (S)	1	2 (S)	4	1 (S)	2	128(R)	256	128(R)	256	128 (R)	256
	M28-01	1 (S)	2	320 (R)	640	0.5 (S)	1	0.5 (S)	1	1 (S)	2	16 (I)	32	320 (R)	640	128 (R)	256
	M 30-01	1 (S)	4	128(R)	256	1 (S)	2	1 (S)	2	1 (S)	2	64 (R)	128	8 (R)	16	128(R)	256
	M36-01	2 (I)	4	128(R)	256	2 (S)	2	2 (S)	2	1 (S)	2	64 (R)	128	128 (R)	256	128 (R)	256
	M43-01	128 (R)	256	128 (R)	256	4 (R)	8	2 (S)	4	1 (S)	2	16 (I)	32	8 (R)	16	64 (R)	256
	M49-02	2 (I)	8	128 (R)	256	2 (S)	4	4 (S)	4	1 (S)	2	128 (R)	256	16 (R)	32	128 (R)	128
	M65-02	1 (S)	4	128 (R)	256	0.5 (S)	1	4 (S)	4	1 (S)	2	64 (R)	64	8 (R)	16	128 (R)	256
	M71-01	4 (R)	16	128 (R)	256	1 (S)	1	1 (S)	2	1 (S)	2	128 (R)	256	128 (R)	256	128 (R)	256
	M90-01	2 (I)	4	64 (R)	128	0.5 (S)	1	1 (S)	2	1 (S)	2	128 (R)	256	128 (R)	256	128 (R)	256
	M91-01	1 (S)	2	128 (R)	256	1 (S)	2	2 (S)	4	2 (S)	2	128(R)	256	16 (R)	16	128 (R)	256
	84	16 (R)	32	64 (R)	128	8 (R)	16	1 (S)	1	0.5 (S)	1	8 (S)	16	32 (R)	32	8 (R)	256
	85	2 (I)	4	128(R)	256	1 (S)	2	0.5 (S)	0.5	0.5 (S)	1	2 (S)	8	4 (I)	8	4 (I)	64
	86	1 (S)	2	128 (R)	256	0.5 (S)	1	0.25(S)	0.5	0.5 (S)	1	32 (R)	64	128 (R)	256	2 (S)	16

**Conjunctivitis USA**	**87**	2 (I)	4	128 (R)	256	0.5 (S)	1	0.5 (S)	1	0.5 (S)	1	2 (S)	4	4 (I)	16	2 (S)	4
	**88**	8 (R)	16	128 (R)	256	1 (S)	2	0.25(S)	1	0.5 (S)	1	2 (S)	4	2 (S)	4	2 (S)	4
	**89**	1 (S)	2	128 (R)	256	0.5 (S)	1	0.5 (S)	1	1 (S)	2	2 (S)	8	8 (R)	16	8 (R)	32
	**90**	64 (R)	128	128 (R)	256	0.5 (S)	1	0.5 (S)	1	1 (S)	1	0.5 (S)	1	16 (R)	32	4 (I)	16
	**91**	1 (S)	2	128 (R)	256	0.5 (S)	1	0.25 (S)	0.5	0.5 (S)	1	1 (S)	2	4 (I)	16	8 (R)	16
	**92**	1 (S)	2	4 (S)	16	0.5 (S)	1	0.5 (S)	1	0.5 (S)	1	2 (S)	4	64 (R)	128	4 (I)	16
	**93**	4 (R)	16	128 (R)	256	1 (S)	2	0.25 (S)	0.5	0.5 (S)	1	2 (S)	4	2 (S)	4	4 (I)	16
	**94**	8 (R)	16	128 (R)	256	2 (S)	2	1 (S)	1	0.5 (S)	1	0.5 (S)	1	4 (I)	4	4 (I)	16
	**95**	16 (R)	32	64 (R)	32	0.5 (S)	1	0.25 (S)	0.5	0.5 (S)	1	1 (S)	2	32 (R)	64	4 (I)	16
	**96**	1 (S)	2	128 (R)	256	1 (S)	2	0.25 (S)	1	0.5 (S)	1	2 (S)	4	4 (I)	16	4 (I)	16
	**97**	0.25 (S)	0.5	0.5 (S)	1	0.5 (S)	1	0.25 (S)	0.5	0.5 (S)	1	0.5 (S)	1	2 (S)	8	4 (I)	16
	**98**	0.25 (S)	0.5	128 (R)	256	1 (S)	2	0.25 (S)	0.5	0.5 (S)	0.5	2 (S)	4	1 (S)	4	4 (I)	16
	**99**	4 (R)	8	128 (R)	256	0.5 (S)	1	0.25 (S)	0.5	0.5 (S)	1	2 (S)	4	4 (I)	16	0.5 (S)	1
	**100**	0.25 (S)	1	128 (R)	256	0.5 (S)	1	0.25 (S)	0.5	0.5 (S)	1	1 (S)	2	4 (I)	16	8 (R)	16
	**101**	128 (R)	256	128 (R)	256	64 (R)	128	0.25 (S)	0.5	0.5 (S)	1	2 (S)	2	64 (R)	128	4 (I)	8
	102	32 (R)	64	32 (R)	64	32 (R)	64	2 (S)	2	0.5 (S)	1	1 (S)	2	4 (I)	8	4 (I)	16
	**103**	32 (R)	64	128 (R)	256	8 (R)	16	0.25 (S)	1	0.5 (S)	0.5	4 (S)	8	32 (R)	64	4 (I)	8
	**104**	128(R)	256	64 (R)	128	128 (R)	256	0.5 (S)	1	0.5 (S)	1	0.5 (S)	1	64 (R)	128	4 (I)	8
	**105**	128 (R)	256	32 (R)	64	32 (R)	64	1 (S)	1	1 (S)	1	0.5 (S)	1	64 (R)	128	32 (R)	64
**Conjunctivitis AUS**	**46**	1 (S)	1	4 (S)	8	1 (S)	1	0.5 (S)	1	1 (S)	1	8 (S)	8	4 (I)	8	4 (I)	8
	**136**	4 (R)	16	16 (R)	32	2 (S)	2	1 (S)	1	0.25 (S)	1	16 (I)	32	8 (R)	32	16 (R)	64
	**134**	1 (S)	2	32 (R)	64	0.5 (S)	1	0.5 (S)	1	0.5 (S)	0.5	4 (S)	4	8 (R)	16	64 (R)	128
	**140**	1 (S)	2	64 (R)	128	0.5 (S)	1	0.5 (S)	1	0.5 (S)	1	8 (S)	16	16 (R)	32	128 (R)	16
**Non-infectious CIE**	**12**	4 (R)	16	64 (R)	128	1 (S)	2	2 (S)	2	1 (S)	1	8 (S)	16	16 (R)	32	4 (I)	8
	**20**	1 (S)	2	32 (R)	64	1 (S)	1	1 (S)	2	0.5 (S)	1	32 (R)	64	16 (R)	32	16 (R)	8
	**24**	0.25 (S)	1	64 (R)	64	0.25 (S)	1	1 (S)	1	0.25 (S)	1	2 (S)	8	4 (I)	16	64 (R)	64
	**25**	1 (S)	2	8 (S)	32	0.5 (S)	2	1 (S)	2	1 (S)	1	8 (S)	16	8 (R)	32	32 (R)	256
	**27**	1 (S)	2	64 (R)	128	8 (R)	16	1 (S)	2	1 (S)	2	2 (S)	8	8 (R)	16	64 (R)	16
	**28**	0.25 (S)	1	4 (S)	8	0.5 (S)	2	0.5 (S)	1	0.5 (S)	2	2 (S)	4	8 (R)	16	4 (I)	16
	**32**	1 (S)	2	2 (S)	4	0.5 (S)	1	2 (S)	2	0.5 (S)	1	4 (S)	8	2 (S)	4	4 (I)	16
	**33**	0.5 (S)	1	2 (S)	4	0.5 (S)	1	0.5 (S)	1	0.5 (S)	1	2 (S)	8	4 (I)	16	2 (S)	4
	**48**	1 (S)	2	16 (R)	32	1 (S)	2	0.5 (S)	1	0.5 (S)	1	16 (R)	32	4 (I)	16	128 (R)	256
	**117**	8 (R)	16	32 (R)	64	2 (S)	4	0.5 (S)	1	1 (S)	2	8 (S)	16	8 (R)	16	128 (R)	256

	**26**	0.5 (S)	1	64 (R)	128	0.25 (S)	0.5	0.5 (S)	1	0.5 (S)	0.5	2 (S)	2	64 (R)	128	8 (R)	16
	**29**	1 (S)	2	64 (R)	64	0.5 (S)	1	1 (S)	2	0.5 (S)	1	4 (S)	8	16 (R)	64	128 (R)	256
	**31**	4 (R)	16	16 (R)	32	1 (S)	2	0.5(S)	1	0.5 (S)	1	16 (I)	32	8 (R)	16	128 (R)	256
	**41**	4 (R)	8	64 (R)	128	1 (S)	2	1 (S)	1	1 (S)	2	2 (S)	4	64 (R)	64	4 (I)	8

*, break points for each antibiotic form CLSI and EUCAST. R= resistant, I = Intermediate, S= susceptible. Conj. = conjunctivitis, MK = microbial keratitis, niCIE = non-infectious corneal infiltrative events. Grey shade indicates resistance.
